# The effects of E'Jiao on body composition, bone marrow adiposity and skeletal redox status in ovariectomised rats

**DOI:** 10.7150/ijms.84604

**Published:** 2023-10-09

**Authors:** Sophia Ogechi Ekeuku, Kok-Yong Chin, Jing Qian, Yan Zhang, Haibin Qu, Fairus Ahmad, Sok Kuan Wong, Mohd Mustazil Mohd Noor, Ima Nirwana Soelaiman

**Affiliations:** 1 Department of Pharmacology, Faculty of Medicine, Universiti Kebangsaan Malaysiaa, Kuala Lumpur, Malaysia.; 2 Pharmaceutical Informatics Institute, College of Pharmaceutical Sciences, Zhejiang University, Hangzhou, China.; 3 Department of Anatomy, Faculty of Medicine, Universiti Kebangsaan Malaysia, Kuala Lumpur, Malaysia.

**Keywords:** adipocytes, body composition, fat mass, lean mass, traditional Chinese medicine, oestrogen deficiency

## Abstract

**Background:** Menopause is accompanied by increased oxidative stress, partly contributing to weight gain and bone marrow adiposity. Traditional Chinese medication, E'Jiao, has been demonstrated to reduce excessive bone remodelling during oestrogen deprivation, but its effects on body composition and bone marrow adiposity during menopause remain elusive.

**Objective:** To determine the effects of E'Jiao on body composition, bone marrow adiposity and skeletal redox status in ovariectomised (OVX) rats.

**Methods:** Seven groups of three-month-old female Sprague Dawley rats were established (n=6/group): baseline, sham, OVX control, OVX-treated with low, medium or high-dose E'Jiao (0.26, 0.53, 1.06 g/kg, p.o.) or calcium carbonate (1% in tap water, *ad libitum*). The supplementation was terminated after 8 weeks. Whole-body composition analysis was performed monthly using dual-energy X-ray absorptiometry. Analysis of bone-marrow adipocyte numbers and skeletal antioxidant activities were performed on the femur.

**Results:** Increased total mass, lean mass, and bone marrow adipocyte number were observed in the OVX control versus the sham group. Low-dose E'Jiao supplementation counteracted these changes. Besides, E'Jiao at all doses increased skeletal catalase and superoxide dismutase activities but lowered glutathione levels in the OVX rats. Skeletal malondialdehyde level was not affected by ovariectomy but was lowered with E'Jiao supplementation. However, peroxisome proliferator-activated receptor gamma protein expression was not affected by ovariectomy or any treatment.

**Conclusion:** E'Jiao, especially at the low dose, prevented body composition changes and bone marrow adiposity due to ovariectomy. These changes could be mediated by the antioxidant actions of E'Jiao. It has the potential to be used among postmenopausal women to avoid adiposity.

## Introduction

A decrease in lean mass (LM) and an increase in fat mass (FM) have been observed after the cessation of oestrogen production during menopause 1-3. This observation has been replicated in animal studies, wherein increased body weight and FM are common after ovariectomy due to hyperphagia 4-6. An international study revealed that women lost an average of 0.5 % or 0.2 kg LM annually, but gained 1.7% or 0.45 kg FM annually during menopausal transition 3. Oestrogen deficiency also changes fat distribution 7, subsequently contributing to weight gain. A cross-sectional study in Malaysia reported that 60.5% of the middle-aged and elderly women surveyed had increased waist circumference (≥ 80 cm) 8, highlighting it is a common problem in ageing women.

Since oestrogen upregulates cellular antioxidant enzyme levels 9, its deficiency increases oxidative stress in the body 10. As evidence, circulating biomarkers of oxidative stress were found to be higher in postmenopausal women than in premenopausal women 11. Furthermore, oestrogen has been shown to attenuate oxidative stress in the adipose tissue of female mice 12, suggesting that oxidative stress triggered by oestrogen deficiency may be a significant contributor to increased adiposity and subsequent weight gain. Others demonstrated that oxidative stress upregulated the expression of peroxisome proliferator-activated receptor-gamma (PPARγ), CCAAT/enhancer-binding protein alpha (C/EBPα), and aP2, which are adipogenic markers, in human mesenchymal stem cells and mouse 3T3L1 pre-adipocytes 13.

Besides, bone marrow adiposity occurs following oestrogen deficiency due to ovariectomy or menopause 14. Adipose is a type of heterogeneous tissue. White and brown adipose tissue are functionally different from bone marrow adipose tissue (BMAT), which influences systemic and bone metabolism 15,16. Many illnesses, including osteoporosis, diabetes, and obesity, are linked to bone marrow adipogenesis 15.

Caloric restriction and physical activity are the first-line treatments for weight loss 17. The efficacy of these interventions is hindered by the low compliance of the subjects, and the stringent regime required before noticeable weight reduction 18. The current Food and Drug Administration-approved weight loss drugs for reducing fat absorption (e.g., orlistat) have significant gastrointestinal adverse effects, ranging from nausea, vomiting, stomach aches, diarrhoea and steatorrhea. Centrally acting appetite suppressants, such as phentermine, lorcaserin and bupropion, have been linked to an increased risk of cardiovascular diseases, valvulopathies, sleeplessness, and the development of drug tolerance 19. Although bariatric surgery results in better weight loss in obese patients with a body mass index (BMI) ≥ 35 kg/m^2^, the risks of postoperative or late consequences must be considered 20. Many dietary supplements have been proposed to prevent weight gain, among which calcium, a nutrient associated with bone health, has demonstrated some promising results. A meta-analysis reported that calcium supplementation could reduce body weight in specific populations 21. A low calcium diet has been reported to increase adipogenesis by stimulating the activities of PPARγ and its downstream enzymes, such as glyceraldehyde 3 phosphate dehydrogenase and lipoprotein lipase in rats 22. Many individuals are seeking alternative therapy for weight management, including traditional Chinese medicine (TCM).

Through modern pharmacology research, TCMs have demonstrated various regulatory effects on fat metabolism, ranging from its absorption, synthesis, catabolism and transport, as well as the physiological process of appetite control 23. E'Jiao is made from gelatine of stewed and soaked donkey skin. It is a common TCM with a high collagen content that supports blood nutrient levels, as well as bone and joint health 24,25. A recent study demonstrated that E'Jiao can suppress high bone remodelling in female rats with oestrogen deficiency 26. Another study revealed that E'Jiao could promote haematopoietic stem cells, bone formation units and colony-forming units in mice with myelosuppression 27. The influence of E'Jiao on stem cells' differentiation prompts the hypothesis that it could regulate adipogenesis and body composition. However, evidence on this aspect is lacking. To bridge this research gap, the effects of E'Jiao on weight gain and bone marrow adiposity were investigated in this study using ovariectomised (OVX) rats.

## Materials and methods

### Treatment preparation

Shandong Dong-E-E-Jiao Co., Ltd (Dong-e, China) sponsored the E'Jiao used in the current study. The product sheet indicates that the recommended daily dose of E'Jiao for adults is 6 g (or 0.1 g/kg/day for a 60-kg person). The animal equivalent dose using the body surface ratio 28 was 0.53 g/kg for adult rats (400 g body weight). In this study, 0.26 g/kg (low dose; half the recommended dose), 0.53 g/kg (medium dose; the recommended dose), and 1.06 g/kg (high dose; 2 times the recommended dose) of E'Jiao were given to the rats to establish a dose-dependent effect. Distilled water was used to dissolve the E'Jiao before it was given orally to the animals. Calcium carbonate powder (1 g) was dissolved in 100 mL tap water and given to the positive control group. Calcium carbonate was chosen because it has a higher bioavailability compared to calcium citrate 29.

### Sample size calculation

The sample calculation was performed using G*Power (Universität Düsseldorf, Düsseldorf, Germany) based on the difference in fat mass two months post-surgery between sham and OVX rats in a similar study 30. The following values were entered into the software: tails=2; effect size=2.07; alpha error probability=0.5; power=0.8 and ratio between groups=1. The final calculated sample size for each group was 5 per group but we added 1 rat in anticipation of unexpected death during the experiment.

### Animals

Approval from the Animal Ethics Committee of Universiti Kebangsaan Malaysia was obtained before the start of the experiment (Approval code: FAR/FP/KOK YONG/25-MAR./1088-MAR.-2020-FEB.- 2022).

Female Sprague-Dawley rats (*n*=42, aged three months old) were sourced from the Laboratory Animal Resource Unit, Universiti Kebangsaan Malaysia, and housed under the regular settings of the laboratory (temperature 27°C; 8:16 light-to-dark cycle; free access to tap water and food). After conditioning for 7 days, 3 groups of rats were created, i.e. baseline (*n*=6), sham (*n*=6), and OVX groups (*n*=30). The OVX rats were assigned randomly to groups that received calcium carbonate, low, medium, or high doses of E'Jiao, as well as distilled water (as a negative or OVX control). The baseline was sacrificed in the absence of interventions. The OVX groups underwent bilateral ovariectomy while the sham group underwent laparotomy without ovary removal. The supplementation was initiated 7 days post-surgery. The sham and negative control groups received tap water, the calcium-supplemented groups received 1 % calcium carbonate (w/v) in tap water, whereas the E'Jiao-supplemented groups received E'Jiao solution at 0.26 (low), 0.53 (medium), and 1.06 (high) g/kg/day via oral gavage. Body composition was assessed at baseline (month 0), one month and two months later. The rats were euthanized after two months of treatment. The left and right tibias were stored at -80°C for subsequent study, while the left and right femurs were extracted and preserved in 10% neutral buffered formalin.

### Body composition assessment

Discovery Wi Dual-energy X-ray Densitometer (DXA; Hologic, MA, USA) was used to determine the body composition of the rats. The whole-body scans were analysed using 13.5.3 analysis software 31. The rats were sedated with ketamine/xylazine/Zoletil cocktails (0.1 mL/100 g) and placed ventrally on the scanning table. The parameters derived were total body mass (TBM), fat mass (FM), fat percentage (FP), and lean mass (LM).

### Bone histomorphometry

The left femur was divided into halves longitudinally after being stripped of soft tissue. A portion of the bone tissue was decalcified at ambient temperature for 30 days using 10% ethylenediaminetetraacetic acid (EDTA). Following decalcification, the femur was embedded in paraffin and cut into sections of 5 µm thickness with a microtome (Leica RM2235, Nussloch, Germany). To deparaffinise the tissue, the sections were rinsed with xylene and rehydrated with decreasing strength of alcohol. Haematoxylin and eosin were used to stain the rehydrated tissues. Subsequently, dehydration of the sections with increasing concentrations of alcohol and xylene was performed. A light microscope (Zeiss Primo Star, Germany) was used to examine the slides. The images were acquired at a magnification of 100 using the Zen 2.6 lite software.

The remaining half of the femur underwent 72 hours of decalcification in 10% EDTA at 37°C. After rinsing in distilled water, they were kept overnight at -80°C. Next, tissues were embedded in an optimal cutting temperature compound. The cryostat microtome (Thermo ScientificTM HM525 NX Cryostat, UK) was used to section the frozen femur tissues into 5 µm thick sections, which were then put on Polysine® glass slides. The sections were stained with Oil Red O dye (Sigma-Aldrich, St. Louis, MO, US), for 10 minutes, and then counterstained with hematoxylin. A light microscope was used to capture the images (Olympus BX53, Tokyo, Japan). Using ImageJ 1.52a software (National Institutes of Health, USA), the red colour-stained area was analysed to determine the number of adipocytes per tissue area (N.Ac/T.Ar).

### Skeletal PPAR-γ levels

The left tibia was ground to powder with liquid nitrogen and the protein content was extracted using RIPA buffer. Using the Bradford test, the lysate's total protein content was measured. PPAR-γ levels in the bone were measured using an enzyme-linked immunoassay (Cat number: E-EL-R072; Lot number: AK11V4V665) per the manufacturer's instructions. The protein input in each well was standardised to 2 mg/mL.

### Skeletal redox status

#### Sample preparation

The samples were prepared following the protocols by Ekeuku et al., (2015). Each 0.5 g of right tibia tissue was macerated in 5 mL of 0.15 M Tris buffer (pH 7) or 3 mL of 0.15 M phosphate buffer (pH 7.4) respectively. The phosphate-buffered homogenate was centrifuged for an hour at 3000 rpm at 4°C. The Tris buffer homogenate was centrifuged for 30 minutes at 4°C. After being collected, the supernatant was stored at -80°C.

#### Total protein level estimation

The total protein content of each sample was calculated using the Bradford method with bovine serum albumin standards. A 96-well microplate was quickly filled with 290 µL of Bradford reagent. Following the addition of 10 µL of phosphate or Tris bone tissue homogenate, The mixture's absorbance was measured at 595 nm, after being stirred and incubated for 5 minutes at room temperature.

#### Glutathione (GSH) assay

The procedure was modified from Ekeuku et al., (2015). Briefly, 60 µL of Tris homogenate was added to a 96-well plate, followed by 30 µL of Ellman's reagent (19.8 mg 5,5'-dithio-bis-(2-nitrobenzoic acid)/0.1% sodium nitrate). Subsequently, 180 µL of 0.2 M phosphate buffer (pH 8) was incorporated into the mixture. The content of the wells was thoroughly mixed and incubated for 5 minutes at 25°C. The absorbance of the mixture was measured at 412 nm. GSH concentration was represented as mmol/mg protein.

#### Superoxide dismutase (SOD) assay

The procedures were modified from the studies by Ekeuku et al., (2015) and Khare et al., (2019). Briefly, 60 µL of 50 mM sodium carbonate, 24 µL of 25 M nitroblue tetrazolium, 12 µL of 0.1 mM EDTA, and 30 µL of Tris tissue homogenate were mixed in a 96-well plate. After that, 48 µL of 1 mM hydroxylamine hydrochloride was incorporated into the mixture. After 2 minutes, the absorbance of the mixture was determined at 560 nm. SOD activity was reported as U/mg protein.

#### Catalase (CAT) assay

The catalase assay was carried out with minor modifications as per Ekeuku et al., (2015) and Khare et al., (2019) with slight modifications. Briefly, 190 µL of 0.05 M phosphate buffer (pH 8), 10 µL of phosphate-buffered bone tissue homogenate and 100 µL of 0.03 M hydrogen peroxide were added into a 96-well plate. The absorbance of the mixture was recorded at 612 nm every 30 seconds for 2 mins. Catalase activity was represented as mmol/mg of protein.

#### Malondialdehyde (MDA) assay

Lipid peroxidation was quantified following the study by Ekeuku et al., (2015) with minor changes. Briefly, 40 µL of 10% trichloroacetic acid, 20 µL of 0.9% saline (w/v), and 20 µL of Tris homogenate were combined in a 2 mL microcentrifuge tube. The samples were centrifuged at 3000 rpm and 25^°^C for 10 minutes. A microcentrifuge tube holding 10 µL of 1% thiobarbituric acid received a total of 40 µL of supernatant. The tubes were immersed in a 95°C water bath for one hour of incubation. The test tubes were taken out of the water bath, and their contents were transferred to a 96-well plate. The absorbance of the mixture was read at 532 nm. The levels of MDA were expressed as nmol/g wet tissue.

### Statistical analysis

The statistical analysis was performed using Statistical Package for Social Sciences version 23.0 (IBM Armonk, USA). The Shapiro-Wilk test was employed to determine whether the data were normal. In analysing normally distributed data with a time×group design, mixed-design analysis of variance (ANOVA) with small effect analysis was performed. To analyse normally distributed data with endpoint design, one-way ANOVA was utilised along with Tukey's post hoc test. Skewed data were analysed using the Kruskal Wallis test and the Mann-Whitney U-test with Bonferroni correction. Data that were normally distributed were represented using the mean and standard error of the mean (SEM). The skewed data were depicted using the median and interquartile range (IQR). Statistical significance was defined as a p-value<0.05

## Results

### Body composition assessment

**Figure 1:**
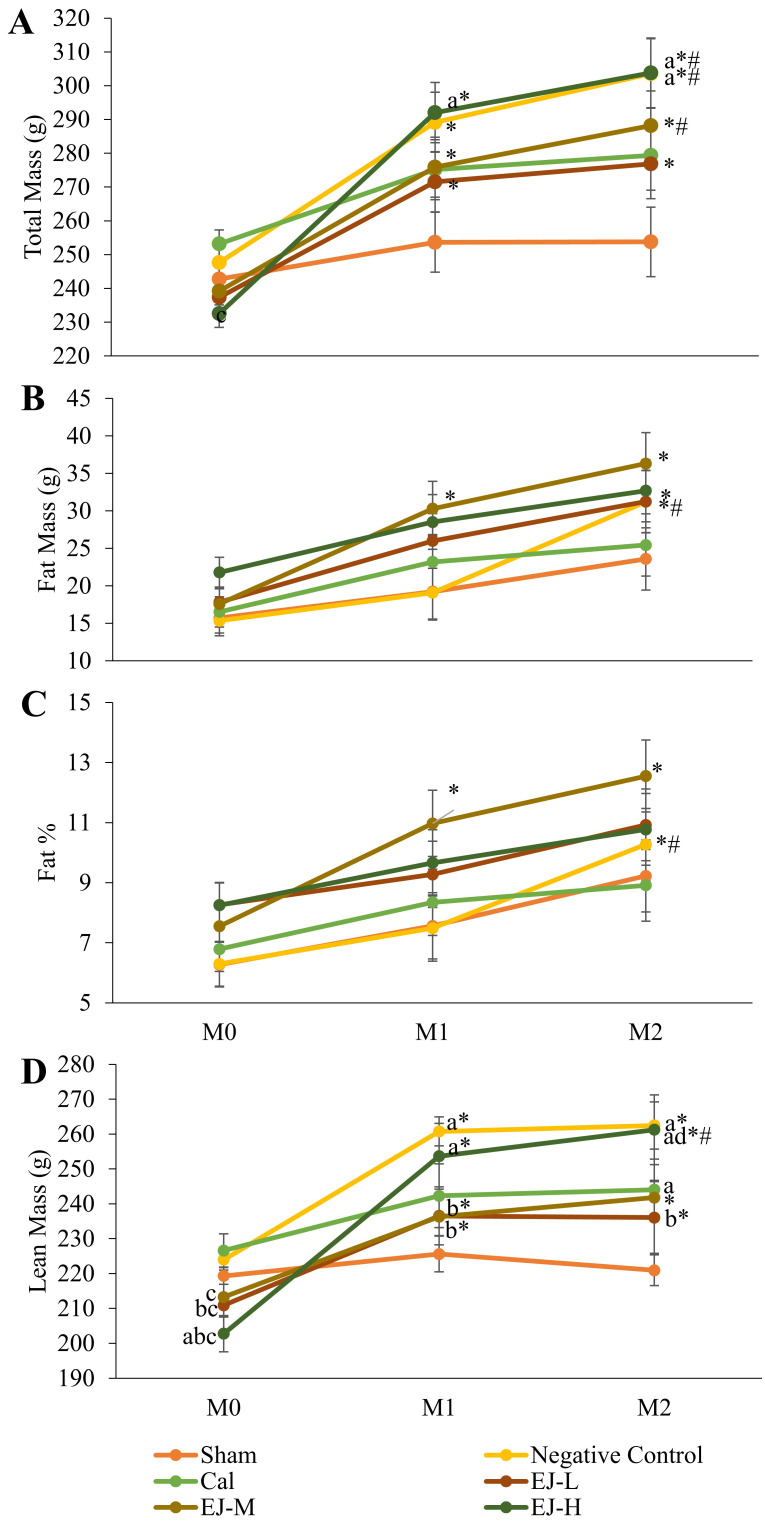
Body composition analysis of the rats during the study, showing total body mass (A), fat mass (B), fat percentage (C), and lean mass (D). The data were reported as mean ± standard error mean (*n*=6/group). Using mixed-design ANOVA and Tukey post hoc pairwise comparison, statistical significance was assessed. A significant difference (*p* < 0.05) is denoted by 'a' vs the sham; 'b' vs negative control; 'C' vs calcium; 'd' vs EJ-L; 'e' vs EJ-M at the same time point; * vs M0 within the same group; # vs M1 within the same group. Abbreviations: Cal, calcium carbonate; EJ-L, E'Jiao -low dose; EJ -M, E'Jiao -medium dose; EJ -H, E'Jiao -high dose; M0, month 0; M1, month 1; M2, month 2; %, percentage.

Except for the control and calcium carbonate groups, all groups demonstrated a significant rise in TBM over time. TBM significantly increased in month 1 and 2 compared to month 0 in the negative control group (p<0.001 at both time points) and E'Jiao supplemented groups (low: p=0.002 at both time points; medium: p=0.001 in month 1, p=0.001 in month 2; high: p=0.001 at both time points). TBM was significantly higher in the medium- (p=0.002) and high-dose E'Jiao supplemented groups (p=0.004) as well as the negative control group (p=0.001) at month 2 compared to month 1. The between-group comparison revealed that TBM was significantly higher in the negative control group in month 2 compared to the sham group (p=0.014), and in the high-dose E'Jiao supplement group in month 1 and 2 compared to the sham group (p=0.043 and p=0.014) (Figure 1A).

In the negative control, a significant time-dependent increase in FM was observed in month 2 versus month 0 (p=0.0047) and month 1 (p<0.001). FM increased in the low-dose E'Jiao-supplemented group in month 2 compared to month 0 (p=0.019). There was a time-dependent increase in FM for the medium-dose E'Jiao-supplemented group in month 1 and 2 compared to month 0 (p=0.027 and p<0.001, respectively). At each time point, there was no discernible variation in FM across all study groups (p>0.05). (Figure 1B).

A significant time-dependent increase in FP was seen in the negative control in month 2 compared to month 0 (p=0.022) and month 1 (p=0.004). In addition, the rats supplemented with medium-dose E'Jiao demonstrated a time-dependent rise in FP from month 0 to month 1 and 2 (p=0.049 and 0.003, respectively). At each time point, there was no discernible variation in FP across all study groups (p>0.05) (Figure 1C).

Except for the sham and calcium-supplemented groups, all groups displayed a significant rise in LM over time. In comparison to month 0, there was a significant time-dependent increase in LM in the E'Jiao-supplemented groups (low: p=0.003 in month 1 and p=0.006 in month 2; medium: p=0.006 in month 1 and p=0.002 in month 2; high: p<0.001 at both time points) and negative control (p<0.001 at both time points). A time-dependent increase in LM was also seen in the high-dose E'Jiao-supplemented group in month 2 compared to month 0 (p=0.025). The between-group comparison revealed that LM was significantly higher in the high-dose E'Jiao-supplemented group than in the sham group in month 0 (p=0.008). Low- (p=0.034) and high-dose E'Jiao supplementation reduced LM in month 0 when compared to the negative control (p=0.001). In month 0, the E'Jiao-supplemented groups displayed decreased LM in comparison to the calcium-supplemented group (E'Jiao -L: p=0.012; E'Jiao-M: p=0.031; and E'Jiao-H: p=0.0002). In month 1, the low and medium-dose E'Jiao-supplemented groups showed significantly lower LM compared to the negative control (p=0.022 and p=0.021), while the high-dose E'Jiao-supplemented group showed significantly higher LM compared to the sham (p=0.001 and p=0.009). In month 2, the calcium-supplemented, high-dose E'Jiao-supplemented, and negative control groups all displayed higher LM compared to the sham group (p=0.001, 0.044, and 0.001, respectively). The high-dose E'Jiao-supplemented group had LM that was significantly higher than the low-dose E'Jiao-supplemented group at the same time point (p=0.029), while the low-dose E'Jiao-supplemented group had LM that was significantly lower than the negative control (p=0.023) (Figure 1D).

### Bone marrow adiposity and PPAR-γ expression

**Figure 2:**
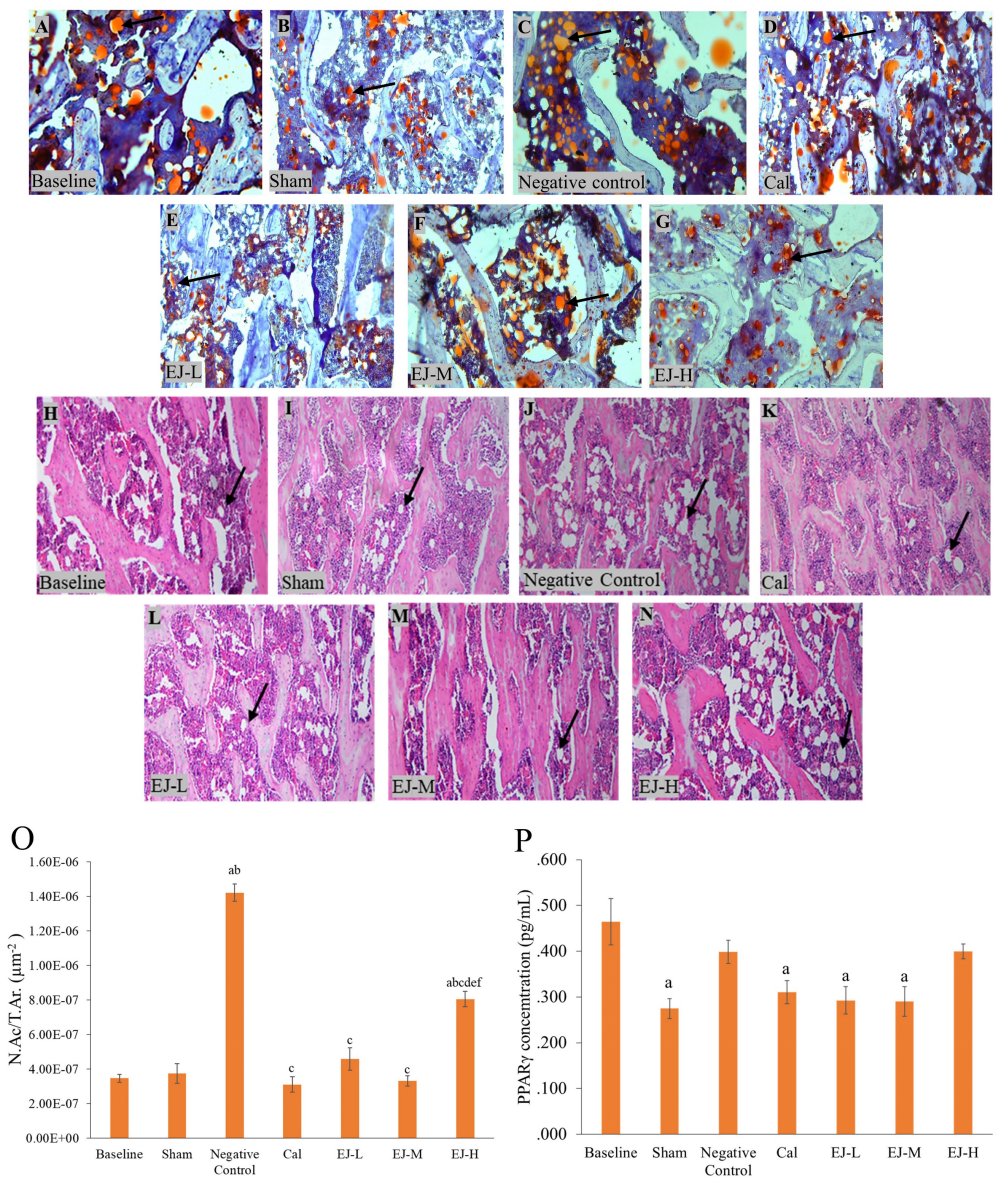
Micrograph of femur sections stained with oil red O (A-G) at 100× and with haematoxylin and eosin (H-N) at 100×. PPAR-γ level (P) and the number of adipose cells (O) in the rat femur's bone marrow are measured. The data are reported as mean standard error mean (*n*=6/group). One-way ANOVA with Tukey post hoc pairwise comparison was used to assess the statistical significance. The letter 'a' denotes a significant difference (p<0.05) vs baseline, 'b' vs sham, 'c' vs negative control, 'd' vs calcium, 'e' vs EJ-L, and 'f' vs EJ-M. The arrows indicate possible adipocytes in the bone marrow. Abbreviations: Cal, calcium; EJ-L, E'Jiao-low dose; EJ-M, E'Jiao-medium dose; EJ-H, E'Jiao-high dose; N.Ac./T.Ar., number of adipose cells per tissue area; PPAR-γ, peroxisome proliferator activator gamma.

Multiple lipid droplet shapes were visible in the OVX rats' bone marrow after haematoxylin and eosin staining. Oil red O staining, which gave the droplets a red colour, confirmed this observation (Figure 2). When compared to the baseline and the sham group, the rats in the negative control group and those receiving high-dose E'Jiao supplementation had higher N.Ac./T.Ar (p<0.001 for all comparisons). Compared to the negative control and high-dose E'Jiao-supplemented groups, the calcium, low- and medium-dose E'Jiao-supplemented groups revealed a significant decrease in N.Ac./T.Ar (p<0.001 for all comparisons) (Figure 2O).

### PPAR-γ protein level

The PPAR-γ protein level was lower in the sham group versus the baseline group (p=0.002). However, the PPAR-γ protein level was unaffected by ovariectomy. Rats treated with calcium (p=0.017), low-(p=0.006) and medium-dose E'Jiao (p=0.005) also showed decreased PPAR-γ protein levels compared to the baseline group, but not with OVX control (p>0.05) (Figure 2P).

### Skeletal markers of redox status

**Figure 3:**
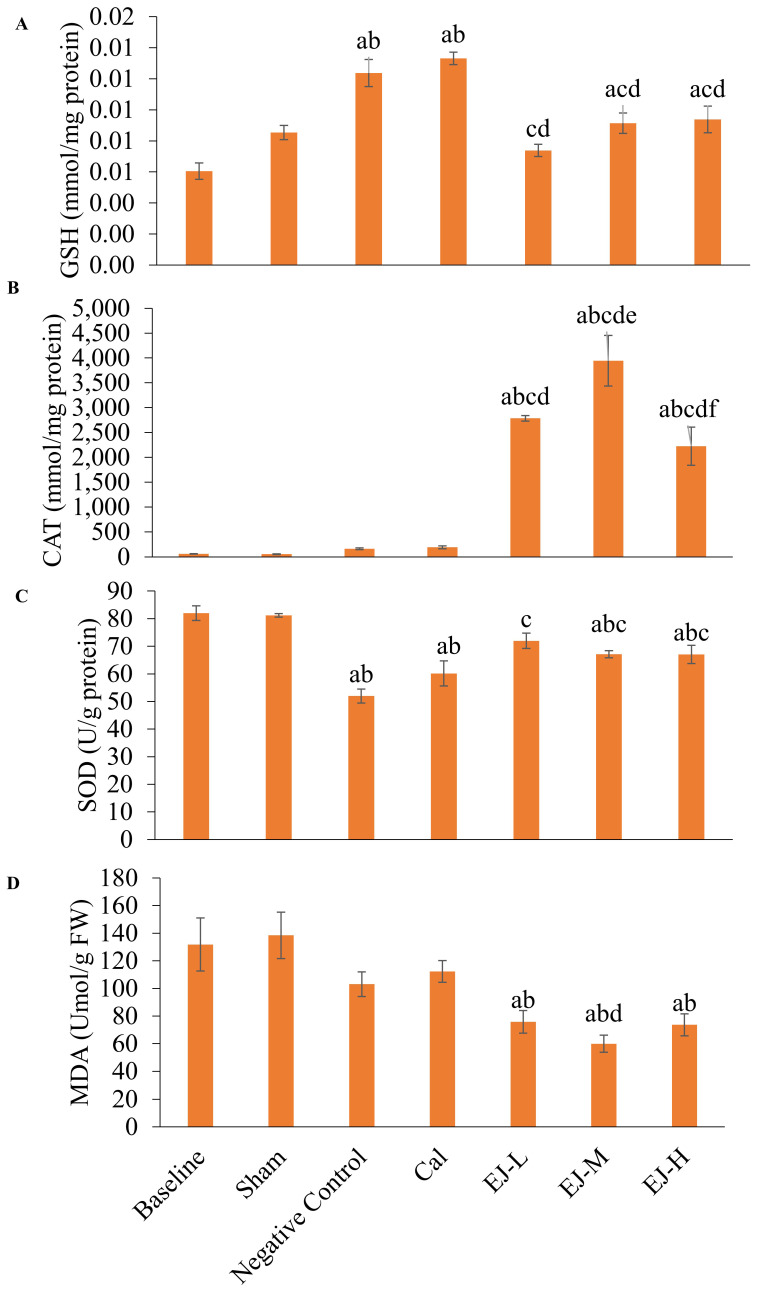
The redox status of the rats is reflected by GSH level (A), CAT activities (B), SOD level (C) and MDA level (D). The data are reported as mean ± standard error mean (*n*=6/group). One-way ANOVA with Tukey post hoc pairwise comparisons was used to assess the statistical significance. The letter 'a' denotes a significant difference (p<0.05) vs 'a' baseline, 'b' sham, 'c' negative control, 'd' vs calcium, 'e' vs EJ-L and 'f' vs EJ-M. Abbreviations: Cal, calcium; EJ-L, E'Jiao-low dose; EJ-M, E'Jiao-medium dose; EJ-H, E'Jiao-high dose; FW, femur's weight; GSH, glutathione; CAT, catalase; SOD, superoxide dismutase; MDA, malondialdehyde.

Compared to the baseline and the sham group, GSH level was higher in the calcium-supplemented group (versus baseline: p<0.001; sham: p=0.001) and negative control group (versus baseline: p<0.001; sham: p=0.001). In comparison to the negative control and calcium-supplemented group, treatment with low- (p<0.001 for all comparisons), medium- (p=0.010; calcium carbonate: p=0.004), and high-dose E'Jiao (p=0.021; calcium carbonate: p=0.008) reduced GSH level in the rats. In comparison to the baseline group, GSH levels were higher in the medium- (p=0.016) and high-dose E'Jiao-supplemented groups (p=0.007) (Figure 3A).

No significant change in CAT activities was observed after OVX induction and calcium treatment (versus the baseline and sham group). However, treatment with low-, medium- and high-dose E'Jiao significantly increased CAT activities in OVX rats in comparison to baseline, sham, negative control and calcium carbonate group (p<0.001 for all comparisons) (Figure 3B).

SOD level was reduced in the negative control (p<0.001 for both comparisons), calcium (p<0.001 for both comparisons), medium- (baseline: p=0.010; sham: p=0.018) and high-dose E' Jiao groups (baseline: p=0.010; sham: p=0.017) compared to the baseline and sham groups. However, low- (p<0.001), medium- (p=0.008) and high-dose E'Jiao (p=0.009) increased SOD levels in OVX rats (Figure 3C).

MDA level was not affected by OVX. However, E'Jiao treatment at all doses reduced MDA levels compared to baseline (low: p=0.027; medium: p=0.002; high: p=0.019) and sham (low: p=0.010; medium: p=0.001; high: p=0.007). The MDA level of rats supplemented with medium-dose E'Jiao (p=0.046) was lower compared to rats supplemented with calcium (Figure 3D).

## Discussion

The current study demonstrated that TBM, LM, FM, and FP levels increased with time in the OVX groups, despite a lack of significant difference in FM and FP compared with the sham group. In addition, bone marrow adiposity increased with ovariectomy. E'Jiao at a low dose, however, suppressed the rise in LM. Low- and medium-dose E'Jiao also prevented bone marrow adiposity. Increased CAT activities and SOD levels were noted in groups treated with E'Jiao. GSH level was increased in the OVX group, maybe due to a physiological response to increased oxidative stress, but it was reduced with E'Jiao. However, E'Jiao did not affect FM and FP significantly in OVX rats. The skeletal PPARγ levels did not change significantly with ovariectomy and treatments.

DXA is a validated imaging method to assess body composition. It can accurately characterise LM and FM, as well as bone mineral density in rodents and humans 34-36. In the present study, the OVX rats had significantly higher TM and LM than the sham group 8 weeks after castration. The OVX rats also experienced a significant time-dependent increase in FM and fat percentage, which was not experienced by the sham group. Avelin et al. reported that FM and LM increased in OVX Wistar rats significantly versus after 8 weeks 6. Similarly, Chen and Haiman showed that FM and LM increased significantly in OVX Sprague-Dawley rats versus the sham groups 35 days after the surgery 37. Thus, the changes in body composition in the OVX rats in this study agreed with the previous studies. Despite the lack of effects on TM, FM and FP, low- and medium-dose E'Jiao suppressed OVX-induced increase in LM. The exact mechanism for this observation was not known at this moment, since the food intake and energy metabolism of the rats were not determined.

The physiological function of BMAT remains elusive but it is morphologically similar to white adipose tissue. They can secrete adipokines, inflammatory cytokines and receptor activator of nuclear factor kappa- Β ligand (pro-osteoclastogenesis factor) 38. However, it has been demonstrated that they are resistant to lipolysis and insulin actions 39. Under pathophysiological conditions, expansion of BMAT has been observed with ageing, osteoporosis, anorexia nervosa and obesity 40. Recent studies also highlighted that BMAT might shape the bone microenvironment for cancer progression 41.

Oestrogen deficiency has been found to increase BMAT 42,43. Several reports have demonstrated increased bone marrow adiposity in female rats 44,45 and rabbits 46 following castration. The present study concurred with the previous studies, wherein the OVX rats had high marrow adiposity evidenced by increased N.Ac/T.Ar. in comparison to the sham group. Low-, medium- and high-dose E'Jiao suppressed the increase in N.Ac/T.Ar in OVX rats, suggesting that it could prevent bone marrow adiposity.

PPAR-γ is a nuclear hormone receptor that is essential for adipocyte differentiation 47. PPAR-γ activation has been shown to stimulate adipogenesis and BMAT formation 48. As a result, inhibiting PPAR-γ expression appears to be a logical approach to preventing bone marrow adiposity after menopause 49. In this study, skeletal PPARγ levels were not altered by ovariectomy and treatments significantly. It has been demonstrated that E'Jiao promotes the development of hematopoietic stem cells, hematopoietic progenitor cells, and bone marrow nucleated cells 27. However, its effects on mesenchymal stem cell differentiation, where adipocytes are derived, have not been determined. Thus, E'Jiao could modulate bone marrow adipogenesis through mechanisms other than regulating PPARγ expression.

Antioxidants could regulate bone marrow adiposity by inhibiting adipogenesis and increasing adipocyte apoptosis derived from human bone marrow mesenchymal stromal cells 50,51. E'Jiao possesses antioxidant effects, wherein it reduced oxidative stress in D-galactose-induced ageing mice by increasing SOD, CAT and glutathione peroxidase activities, while reducing MDA levels 52. Similarly, in the present study, E' Jiao supplementation increased SOD levels and CAT activities, while reducing MDA levels. GSH levels were increased by OVX but decreased by E'Jiao supplementation. The increase in GSH levels may be a physiological response to the increased oxidative stress due to oestrogen deficiency, which was prevented by E'Jiao. This observation indicates that E'Jiao's effects in suppressing bone marrow adiposity could be partially attributed to its antioxidant potential.

In the present study, calcium supplementation reduced bone marrow adiposity but did not affect body composition and skeletal PPAR-γ level compared to the negative control group. Goudarzi et al. (2018) reported calcium inhibited the differentiation of adipose-derived mesenchymal stem cells into adipocytes, implying that calcium may be exerting an inhibitory effect on adipogenesis 53. This is supported by in vitro studies reporting the inhibitory effects of calcium or calcium channel regulators on adipogenic differentiation in murine and human preadipocytes 54-57. However, several other studies have reported reduced PPAR-γ levels, fat deposition and fat content following calcium supplementation 56,58,59, which was not shown in the current study. The exact reason for the discrepancy is not known.

Several limitations of this study should be noted. In rats, a lack of oestrogen frequently causes hyperphagia and weight gain 60, but food intake was not measured in this study. The effects of castration and treatments on visceral fats, adipokines and anti-/pro-inflammatory cytokines of physiological importance were not determined. The effects of E'Jiao and calcium could be more obvious with an increased treatment period. Both agents were not supplemented in uncastrated animals to investigate their effects under a physiological level of oestrogen. Future studies should also use biochemical methods to support the changes in body composition and BMAT in ovariectomised rats. These aspects could be considered in future studies. Nonetheless, this study presents preliminary data on E'Jiao as a therapeutic agent for increased bone marrow adiposity associated with oestrogen deficiency.

## Conclusion

E'Jiao improved antioxidant status and decreased oxidative stress in OVX rats, which prevented the growth in body mass and bone marrow adiposity. The potential use of E'Jiao in this regard can reduce the healthcare burden due to adiposity, such as cardiovascular diseases and diabetes mellitus. However, to verify this impact, a thorough investigation into the mechanism by which E'Jiao controls adipocyte differentiation will be necessary. A well-planned clinical trial would also be required to validate the effects of E'Jiao in postmenopausal women.
